# Interventions to improve antimicrobial prescribing of doctors in training: the IMPACT (IMProving Antimicrobial presCribing of doctors in Training) realist review

**DOI:** 10.1136/bmjopen-2015-009059

**Published:** 2015-10-22

**Authors:** Geoff Wong, Nicola Brennan, Karen Mattick, Mark Pearson, Simon Briscoe, Chrysanthi Papoutsi

**Affiliations:** 1 Nuffield Department of Primary Care Health Sciences, University of Oxford, Oxford, UK; 2 Collaboration for the Advancement of Medical Education Research Assessment, Plymouth University Peninsula Schools of Medicine and Dentistry, Plymouth, UK; 3 University of Exeter Medical School, University of Exeter, Exeter, UK; 4 Collaboration for Leadership in Applied Health Research and Care (CLAHRC) for the South West Peninsula, University of Exeter Medical School, Exeter, UK

**Keywords:** EDUCATION & TRAINING (see Medical Education & Training), INFECTIOUS DISEASES, MICROBIOLOGY

## Abstract

**Introduction:**

Antimicrobial resistance has been described as a global crisis—more prudent prescribing is part of the solution. Behaviour change interventions are needed to improve prescribing practice. Presently, the literature documents that context impacts on prescribing decisions, yet insufficient evidence exists to enable researchers and policymakers to determine how local tailoring should take place. Doctors in training are an important group to study, being numerically the largest group of prescribers in UK hospitals. Unfortunately very few interventions specifically targeted this group.

**Methods and analysis:**

Our project aims to understand how interventions to change antimicrobial prescribing behaviours of doctors in training produce their effects. We will recruit a project stakeholder group to advise us throughout. We will synthesise the literature using the realist review approach—a form of theory-driven interpretive systematic review approach often used to make sense of complex interventions. Interventions to improve antimicrobial prescribing behaviours are complex—they are context dependent, have long implementation chains, multiple non-linear interactions, emergence and depend on human agency. Our review will iteratively progress through 5 steps: step 1—Locate existing theories; step 2—Search for evidence; step 3—Article selection; step 4—Extracting and organising data; and step 5—Synthesising the evidence and drawing conclusions. Data analysis will use a realist logic of analysis to describe and explain what works, for whom, in what circumstances, in what respects, how and why to improve antimicrobial prescribing behaviour of doctors in training.

**Ethics and dissemination:**

Ethical approval was not required for our review. Our dissemination strategy will be participatory and involve input from our stakeholder group. Tailored project outputs will be targeted at 3 audiences: (1) doctors in training; (2) clinical supervisors/trainers and medical educators; and (3) policy, decision makers, regulators and royal societies.

## Background

### Introduction

A post-antibiotic era—in which common infections and minor injuries can kill—far from being an apocalyptic fantasy, is instead a very real possibility for the 21st century—Dr Keiji Fukuda, World Health Organisation (WHO) Assistant Director-General (1).

Antimicrobial resistance (AMR) is the ability of microorganisms that cause disease to withstand attack by antimicrobial medicines. AMR is also costly, estimated at US$21–US$34 billion per annum in the USA.^1^ The consequences extend beyond patients who present with infections. Many surgical procedures (where antibiotics are given prophylactically in the hope of preventing infections) may be harder to justify as the risk and consequences of infection become more likely and serious.

There are two important strands of activity to address this global crisis. The first is to develop new antimicrobials, the second is to urge all countries to be more prudent in their use of antimicrobials—this is the focus of our proposed research. Antimicrobial stewardship aims to promote optimal care for patients with infections, while minimising the public health threat of drug resistance. However, changing prescribing behaviour is difficult and the challenge is significant given that 50% of antibiotic usage in hospitals is inappropriate.^2^

Organisations globally and nationally have responded to the crisis,^3^ for example, WHO Global Strategy for Containment of Antimicrobial Resistance, 2001; World Health Day six-point AMR policy, 2011; Department of Health (DH) UK Antimicrobial Resistance Strategy and Action Plan (2000); DH/British Society of Antimicrobial Chemotherapy's ‘Start Smart, Then Focus’ campaign;^4^ ^5^ and the TARGET Antibiotics toolkit in general practice.^6^ However, these strategies alone cannot address the global crisis at hand. Focused strategic and integrated action, informed by high-quality research targeted at prescribing behaviour, is now required.

### Doctors in training and antimicrobial prescribing

One key element of curtailing the emergence and spread of AMR has been to focus on the prescribing behaviours of healthcare professionals to ensure they are using antimicrobials prudently (eg, right drug, at the right time, at the right dose, for the right duration). The importance of education for prescribing behaviour change has been described as self-evident,^7^ and doctors in training are an important target group, both as numerically the largest prescribers in the hospital setting^8^ and as a key part of a future generation of antimicrobial prescribers. However, the effectiveness of educational interventions has proved variable^9^ due to the complex environments in which these interventions are embedded,^10^ with powerful forces including hierarchy and role modelling dampening the potential effects.

After graduating from medical school in the UK, new doctors enter the 2-year foundation programme, which mostly occurs in hospital settings, before undertaking a further 3–5 years as a core/specialty trainee. All postgraduate doctors are independent prescribers and will prescribe for patients, typically on a daily basis.

Many types of behaviour change interventions have been developed to improve doctors’ antimicrobial prescribing practice—ranging from’ distribution of educational materials,^11–13^ lectures and seminars^1^ ^4^ ^15^ audit and feedback on performance^1^ ^3^ ^16^ to manual and automated reminders.^17^ ^18^ However, very few have been specifically tailored to the needs of doctors in training.

Evidence indicates that it is unclear if current educational prescribing behaviour change interventions have any consistent effect.^19^ For example, two recent systematic reviews found that the impact of particular types or combinations of interventions was highly variable.^9^ ^20^ This raises the question as to why some prescribing behaviour change interventions are successful in some contexts but not in others? Thus, uncertainty exists about which intervention types to implement and if refinements are needed for local circumstances; and if and how existing interventions are instructing doctors in training to prescribe appropriately.

This knowledge gap has partly come about because much of the current literature has not taken sufficient account of the wider context in which doctors in training prescribe antimicrobials. Prescribing is a complex mix of knowledge, skills and behaviours and there is no simple relationship between them.^21^ ^22^ Prescribing the right antibiotic at the right time is not just about having the correct knowledge about local formularies, resistance patterns and dosages, but also understanding a patient's expectations, concerns, comorbidities and social context.

Education is an important element that influences prescribing practice, but it is not the only one. Qualitative work found that the hospital context and processes played important roles.^23^ The antimicrobial prescribing challenges faced by doctors in training ranged from knowledge deficits (not knowing what to do in certain situations), to the mundane of not knowing that local prescribing protocols even existed on a ward, to having to ‘take sides’ when more senior healthcare professionals disagreed on prescribing decisions. This work adds to a growing literature that acknowledges the importance of the wider context. For example, Ross *et al*^10^ point out that doctors in training work within a strict medical hierarchy in complex organisations and their prescriptions are often influenced by other doctors. McLellan *et al*^24^ note that a technical focus on isolated prescribing competencies is unlikely to support doctors in training to become safe prescribers. The implication is that any review that seeks to understand antimicrobial prescribing behaviour change interventions in this group needs to look beyond just educational interventions and seek to make sense of the role of wider contexts. This need to account for context provides the rationale for using realist review methods in this project.

## Methods

### Aim

To understand how interventions to change antimicrobial prescribing behaviours of doctors in training produce their effects.

### Objectives

1. To conduct a realist review to understand how interventions to change antimicrobial prescribing behaviours of doctors in training produce their effects.
2. To provide recommendations on tailoring, implementation and design strategies to improve antimicrobial prescribing behaviour change interventions for doctors in training.

### Review questions

1. What are the mechanisms by which antimicrobial prescribing behaviour change interventions are believed to result in their intended outcomes?
2. What are the important contexts which determine whether the different mechanisms produce intended outcomes?
3. In what circumstances are such interventions likely to be effective?

### Research plans

#### Objective 1: To conduct a realist review

**Figure 1:**
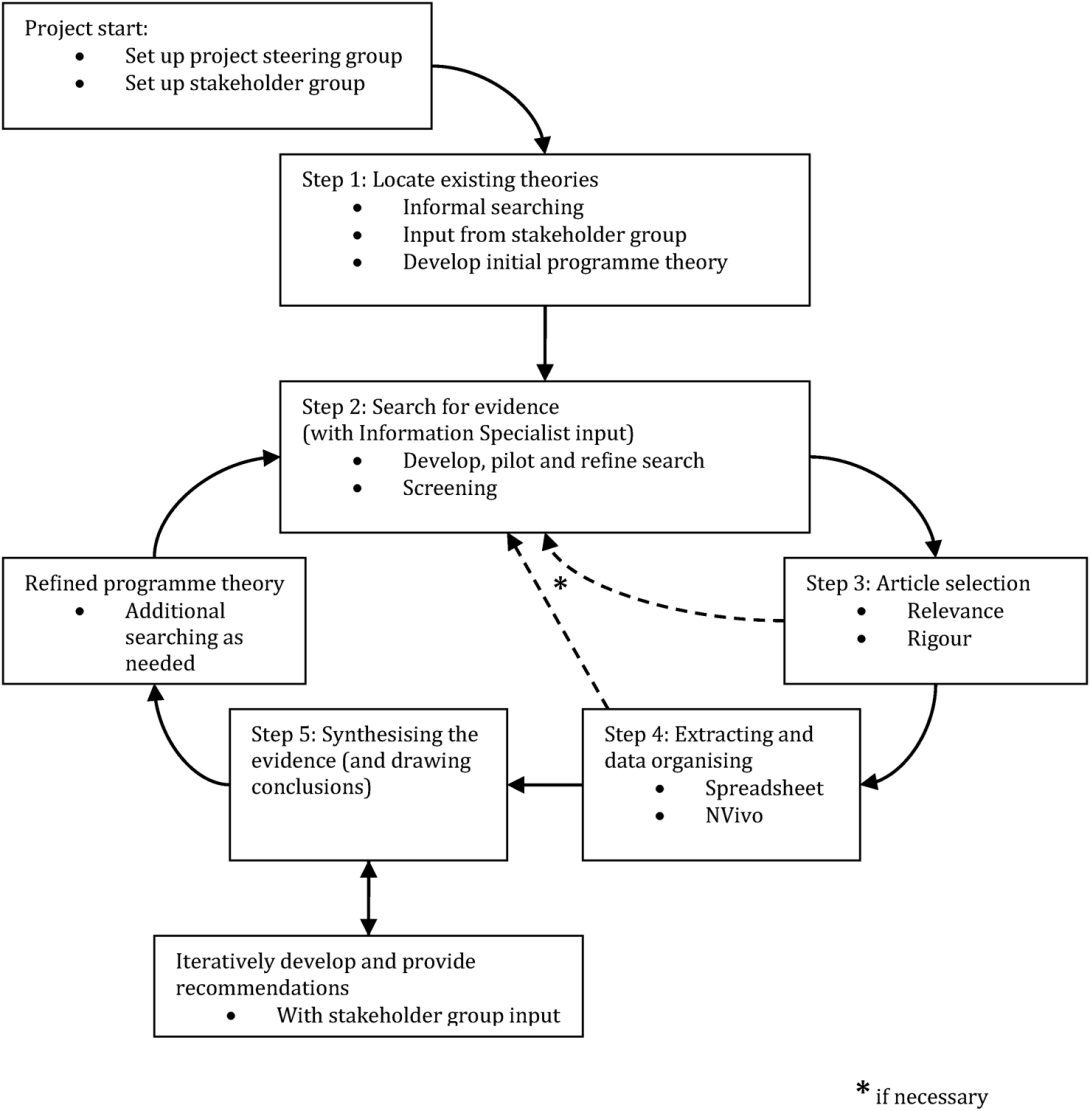
Flow diagram of the project.

Our realist review will be informed by Pawson's five iterative stages.^25^ A pictorial representation of our research plans may be seen in figure 1. We have chosen to use a realist review approach as outlined above in the ‘Introduction’ section. To recap, we have argued that any evidence synthesis that seeks to make sense of how interventions to change antimicrobial prescribing behaviours in doctors in training produce their effects must take into account the context in which the prescribing decisions take place.

The realist review is an interpretive, theory-driven approach to synthesising evidence from qualitative, quantitative and mixed-methods research. Its main strength comes from providing findings that coherently and transferably explain how and why context can influence outcomes.

This process of explanation building starts with the development and refinement of a realist programme theory of interventions to change antimicrobial prescribing behaviours of doctors in training. To do this, we will ‘map’ the sequence of steps needed to achieve the final desired outcome from such an intervention. For each step, a realist logic of analysis will be applied, so as to explain how the (intermediate) outcome for each step might be achieved in realist terms—that is, what interaction between context and mechanism(s) might lead to that outcome. For each step in the sequence, we will seek to identify what mechanism(s) will generate the outcome and in what contexts this mechanism might be triggered. We have defined context as “…any conditions that triggers and/or modifies the behaviour of mechanisms.”^26^ Mechanisms are “…underlying entities, processes, or structures which operate in particular contexts to generate outcomes of interest.” They are usually hidden, sensitive to variations in context and generate outcomes.^27^

Our realist review protocol has been written by the project team and been registered with PROSPERO.^28^ The review will run for an 18-month period from June 2015.

##### Step 1: Locate existing theories

The goal of this step is to identify theories that explain how antimicrobial prescribing behaviour change interventions are supposed to work (and for whom), when they do work, when they do not achieve the desired change in clinical practice, why they are not effective and why they are not being used.^29^ The rationale for this step is that interventions are ‘theories incarnate’—that is underpinning the design of such interventions are theories of why certain components are required. In other words, the designers of interventions have put them together in a certain way based on their theories about what needs to be done to get one or more desired outcomes.^30^ Included within such theories may well be explanations and rationales for process with which an intervention was designed (eg, who designed it, with whom and how?) as these may influence outcomes.^31–33^ For example, the literature indicates that 28% of interventions designed to change antimicrobial prescribing behaviour in new prescribers distribute educational materials.^9^ The theory underlying such a practice is that poor prescribing behaviour is partly due to knowledge deficits, and the way to address this problem is through educational means.

To locate these theories, we will iteratively: (1) consult with key content experts in our stakeholder group (see below), and (2) informally search the literature to identify existing theories. This informal searching differs from the more formal searching process in step 2 in that it is more exploratory and aimed at quickly identifying the range of possible explanatory theories that may be relevant. More exploratory and informal search methods, such as citation tracking and snow-balling,^34^ along with more structured searching for theories^35^ will be used. From these, we will build an initial programme theory to test in the review. Building the programme theory will require iterative discussions within the project team to make sense of and synthesise the different theories into an initial coherent programme theory. We have recruited a stakeholder group (that includes patient and public involvement) to provide content expertise for programme theory refinement and will extend the membership as needed. Once the programme theory has been developed by the project team, it will be presented to the stakeholder group and it will be refined based on their feedback.

##### Step 2: Search for evidence

### Formal search

The purpose of this step is to find a relevant ‘body of literature’ that might contain data with which to further develop and refine the programme theory from step 1. Searching will be designed, piloted and conducted by an information specialist with extensive experience of conducting searches for complex systematic reviews, particularly realist reviews. As a starting point, searching will be guided by the search strategy from a previous closely related systematic review by Brennan and Mattick^9^ on educational interventions to change the behaviour of prescribers in the hospital setting, with a particular emphasis on new prescribers. However, we will need to make modifications to the search strategy used in Brennan and Mattick as this review focused on all prescribers within 2 years of qualification. We will modify the search to focus only on doctors in training. Another modification that will be needed is to increase the scope of the context of the prescribing, as we wish to also include doctors in training who are working in the hospital and primary care. Our preliminary exploratory searching using these modifications indicates that we can expect almost 200 papers that may well be relevant. We anticipate that we may need to search the following bibliographic databases: EMBASE; MEDLINE; CINAHL; PsycINFO; ERIC; DARE; ASSIA, but will be guided by our information specialist (SB). We may also search grey literature sources and any other relevant bibliographic databases identified by our information specialist (SB). Free-text and subject heading search terms will be selected and tested by our information specialist (SB) and the research team using an iterative process to ensure an appropriate balance of sensitivity and specificity. Our information specialist (SB) will translate the search strategies for different databases as required. We will also undertake forwards and backwards citation searches of relevant documents.

Brennan and Mattick's review identified 64 relevant documents, 13 of which specifically focused on doctors in training. The other 51 documents had large numbers of participants (including doctors) from all stages of training and will likely contain some data that are relevant. By increasing the scope to include both hospital and primary care for our realist review, we are confident there are sufficient documents to form a ‘body of literature’ with which to refine any initial programme theory we develop.

### Screening

For the initial search above (step 1: Locate existing theories), our inclusion and exclusion criteria will be broad as we seek to find quantitative, qualitative and mixed-methods documents. For the purposes of this review, prescribing will be defined as the act of determining what medication a patient should have and the correct dosage and duration of treatment.^9^ The following criteria will be applied:

Inclusion:

- Aspect of prescribing—all studies that focused on developing one or more aspects of prescribing as defined above.
- Study design—all study designs.
- Types of settings—all studies that were conducted in hospital or primary care settings.
- Types of participants—all studies that included doctors in training (any specialty and at any level). If the study participants involved all prescribers in a hospital setting (which would include doctors in training) then it will be included.
- Types of intervention—interventions or resources that focus on changing or developing antimicrobial prescribing behaviour.
- Outcome measures—all prescribing-related outcome measures.

Exclusion:

- Studies focusing only on drug administration.
- Interventions focused primarily on medication counselling or adherence.

Screening will be undertaken by CP. A 10% random subsample of the citations retrieved from searching will be reviewed independently by GW and any disagreements will be recorded and resolved by discussion. If disagreements still remain, then the matter will be resolved by discussion between the whole project team.

### Additional searching

An important process in realist reviews is searching for additional data to inform programme theory development. In other words, more searches will be undertaken if we find that we require more data to develop and test certain subsections of the programme theory. As we have outlined above (in step 1: Locate existing theories) we anticipate that we will need to develop a programme theory that takes into account the influence of the wider context in hospitals and primary care that influence the prescribing behaviour of doctors in training. Areas that we believe that we may need additional searches include the education programme for trainee doctors (including less formal, ward-based teaching and assessments), support systems for doctors in training, conflict resolution, time pressures, dealing with uncertainty, organisational systems and healthcare culture.^23^

These additional search areas will greatly increase the amount of relevant data available to us for the realist review. The review by Brennan and Mattick did not search for such literature as it was beyond the scope of their review. We will therefore have to develop, pilot and refine additional searches with the help of our information specialist (SB). These searches will be more purposive and directed by our emerging programme theory and may require searching in range of different disciplines. Where applicable, we will follow the CLUSTER search method developed by Booth *et al.*^35^

For each additional search, the project team will meet to discuss and set inclusion and exclusion criteria. The screening processes will be as described above for the initial search.

#### Step 3: Article selection

Documents will be selected based on relevance (whether data can contribute to theory building and/or testing) and rigour (whether the methods used to generate the relevant data are credible and trustworthy).^30^ Even when a document found from the initial search has been screened and has met inclusion criteria, it may still not contain any data relevant for programme theory development and refinement.

CP will read all the included papers and finally include documents or studies that contain data relevant to the realist analysis—that is, could inform some aspect of the programme theory. At the point of inclusion based on relevance, an assessment will also be made of rigour (how trustworthy were the data being used).^30^ To illustrate how we will operationalise rigour, if data relevant to an aspect of our programme theory have been generated using a questionnaire, then the trustworthiness of the data would be considered to be greater if: (1) the questionnaire had been previously tested and shown to be reliable and valid; and (2) had not been altered (or if altered subsequent testing followed any alterations). A random sample of 10% of documents will be selected, assessed and discussed by CP and GW to ensure that decisions to finally include have been made consistently and to resolve any differences. The remaining 90% of decisions will be made by CP (though a number of these may require further discussion/joint reading between the CP, GW and/or the wider project team as there may be uncertainty over issues of relevance and/or rigour). We will employ the same decision-making process as outlined above in step 2.

#### Step 4: Extracting and organising data

Data extraction and organising of data will be undertaken by CP. A 10% random subsample of the citations retrieved from searching will be reviewed independently by GW and any disagreements will be recorded and resolved by discussion. If disagreements still remain then the matter will be resolved by discussion between the whole project team.

Full texts of the included papers will be uploaded into NVivo (a qualitative data analysis software tool). Relevant sections of texts relating to contexts, mechanisms and/or their relationships to outcomes will be coded in NVivo. This coding will be both inductive (codes created to categorise data reported in included studies) and deductive (codes created in advance of data extraction and analysis as informed by the initial programme theory). The characteristics of the documents will be extracted separately into an EXCEL spreadsheet. Each new element of data will be used to refine the theory if appropriate, and as the theory is refined, included studies will be rescrutinised to search for data relevant to the revised theory that may have been missed initially.

#### Step 5: Synthesising the evidence and drawing conclusions

Data analysis will use a realist logic of analysis to make sense of the initial programme theory. We will use interpretive cross-case comparison to understand and explain how and why observed outcomes have occurred, for example, by comparing interventions where prescribing behavioural change has been ‘successful’ against those which have not, to understand how context has influenced reported findings.

While the processes with a realist review have been set out above in a linear way, as we have illustrated in figure 1, the review processes are iterative. In addition, once searching has identified potentially relevant full-text articles (ie, at the ‘end’ of step 2), the subsequent steps often take place at approximately the same time. In other words, once the full text of a document has been retrieved and it is being read and assessed for selection (ie, at step 3), analysis and synthesis may start. The purpose of analysis and synthesis is to understand how mechanisms behave under the different contexts described within the documents included in the review. During the detailed assessment for inclusion into the review of any content within a potentially relevant article, a series of questions will be asked and judgements made. These are set out in box 1.

Box 1 **Analysis and synthesis questions and judgements.**

1. *Relevance*:
  - Are the contents of a section of text within an included document referring to data that might be relevant to programme theory development?
2. *Interpretation of meaning*:
  - If it is relevant, do the contents of a section of text provide data that may be interpreted as being context, mechanism or outcome?
3. *Judgements about Context-Mechanism-Outcome- Configurations*:
  - What is the Context-Mechanism-Outcome-Configuration (CMOC) (partial or complete) for the data?
  - Are these data to inform CMOCs contained within this document or other included documents?
  - If so, which other documents?
  - How does this CMOC relate to CMOCs than have already been developed?
4. *Judgements about programme theory*:
  - How does this (full or partial) CMOC relate to the programme theory?
  - Within this same document are there data which inform how the CMOC relates to the programme theory?
  - If not, are these data in other documents? Which ones?
  - In light of this CMOC and any supporting data, does the programme theory need to be changed?
5. *Rigour*:
  - Are the data sufficiently trustworthy and rigorous to warrant making changes to the CMOC?
  - Are the data sufficiently trustworthy and rigorous to warrant making changes to the programme theory?

During the review, we move iteratively between the analysis of particular examples, refinement of programme theory, and further iterative searching for data to test particular subsections of the programme theory. The realist review will follow current quality and publication standards.^36^

#### Objective 2: To provide recommendations

Our programme theory will be used to develop recommendations for improving prescribing behaviour change interventions and their implementation. Further details are provided in the Dissemination and Project outputs section below.

## Dissemination plans

Our dissemination strategy will build on the participatory approach and involve input from our stakeholder group. Our approach will be integrative, valuing the different forms of knowledge needed to produce findings capable of informing complex decision-making.^37^ A range of stakeholders will be interested in the findings and recommendations from our review. Different strategies are likely to be needed. We will draw on the advice and expertise of our stakeholder group to help: (1) clarify who the main players are for dissemination for each audience; and (2) to develop materials which are tailored and relevant to each audience. For each audience, once we have clarified the main players we will contact the organisation directly to seek advice on their preferred channels and format for optimal dissemination to their members. We are aware that there is likely to be overlap in the optimal dissemination strategies for each audience, and our approach will be informed by the ‘Knowledge-to-Action Cycle’—see the Project outputs section below for more details. We anticipate that there will be three main audiences for our project outputs:

- Audience 1: doctors in training;
- Audience 2: clinical supervisors/trainers and medical educators;
- Audience 3: policy, decision makers, regulators and royal societies.

## Project outputs

We want to ensure that the outputs of this project will be useful to the National Health Service (NHS) in the UK. To do this we will use the Knowledge-to-Action Cycle framework provided by the KT Clearinghouse (http://ktclearinghouse.ca/knowledgebase/knowledgetoaction). This is a website that provides knowledge translation resources and is funded by the Canadian Institute of Health Research. The Knowledge-to-Action Cycle graphically sets out the steps necessary in bridging the knowledge-to-action gap. Specifically within this framework, with input from our stakeholder group, this realist review will generate knowledge that will inform the following phases of the Knowledge-to-Action Cycle framework:

- Producing stakeholder relevant knowledge (as described in the ‘knowledge funnel’ in this framework);
- Adapting knowledge to local context; and
- Assessing barriers to knowledge use.

This project will produce three major types of outputs:

1. The findings from the review will be submitted for publication to a high-impact peer-reviewed journal.
  We anticipate that such a publication is most likely to impact at an academic level—informing the understanding and theoretical basis of antimicrobial prescribing behaviour change interventions.
2. A ‘How to’ publication that outlines practical advice to optimise, tailor and implement existing interventions designed to change prescribing behaviour in doctors in training. With this publication we aim to impact the day-to-day antimicrobial prescribing practice of doctors in training. This document is mainly targeted at audience 2: clinical supervisors/trainers and medical educators.
3. User-friendly summaries of the review findings that are tailored to the needs of the following audiences:
  - Doctors in training;
  - Clinical supervisors/trainers and medical educators;
  - Policy, decision makers, regulators and royal societies.

## Discussion

The development of AMR is a pressing issue that healthcare faces around the world. Doctors in training are an important group of prescribers. At present, we have an unclear understanding of the processes that drive the prescribing practice of this group of healthcare professionals and how this might be changed. The literature so far has focused on educational interventions that seek to change their prescribing behaviour, at the expense of considering wider contextual influences. This realist review seeks to inform this debate by looking beyond just educational interventions and into the wider contextual drivers for the prescribing behaviour of doctors in training. This increased understanding of why such behaviour happens will be used to develop recommendations for improving prescribing behaviour change interventions and their implementation. No prior realist review has been undertaken on this or any related topic (eg, prescribing in other healthcare professional groups).

### Importance of the research

AMR is an important topic of international priority. Doctors in training are a large and important group of prescribers, but little is known about what drives their prescribing habits and how these might be changed. This realist review will expand our understanding of this topic area by focusing on contextually relevant explanations and will develop outputs to inform future interventions aiming to change prescribing behaviour among doctors in training.
